# Carotenoids Intake and Cardiovascular Prevention: A Systematic Review

**DOI:** 10.3390/nu16223859

**Published:** 2024-11-12

**Authors:** Sandra Sumalla-Cano, Imanol Eguren-García, Álvaro Lasarte-García, Thomas A. Prola, Raquel Martínez-Díaz, Iñaki Elío

**Affiliations:** 1 Research Group on Foods, Nutritional Biochemistry and Health, Universidad Europea del Atlántico, 39011 Santander, Spain; imanol.eguren@uneatlantico.es (I.E.-G.); alvaro.lasarte@alumnos.uneatlantico.es (Á.L.-G.); raquel.martinez@uneatlantico.es (R.M.-D.); 2 Faculty of Health Sciences, Universidade do Cuanza, Cuito EN250, Bié, Angola; 3 Faculty of Health Sciences, Universidad de La Romana, La Romana 22000, Dominican Republic; 4 Faculty of Social Sciences and Humanities, European University of the Atlantic, 39011 Santander, Spain; thomas.prola@uneatlantico.es; 5 Faculty of Health Sciences, Universidad Internacional Iberoamericana, Arecibo, PR 00613, USA; 6 Department of Health, Nutrition and Sport, Universidad Internacional Iberoamericana, Campeche 24560, Mexico

**Keywords:** cardiovascular diseases (CVDs), carotenoids, systematic review, supplementation, inflammation, prevention

## Abstract

**Background**: Cardiovascular diseases (CVDs) encompass a variety of conditions that affect the heart and blood vessels. Carotenoids, a group of fat-soluble organic pigments synthesized by plants, fungi, algae, and some bacteria, may have a beneficial effect in reducing cardiovascular disease (CVD) risk. This study aims to examine and synthesize current research on the relationship between carotenoids and CVDs. **Methods:** A systematic review was conducted using MEDLINE and the Cochrane Library to identify relevant studies on the efficacy of carotenoid supplementation for CVD prevention. Interventional analytical studies (randomized and non-randomized clinical trials) published in English from January 2011 to February 2024 were included. **Results:** A total of 38 studies were included in the qualitative analysis. Of these, 17 epidemiological studies assessed the relationship between carotenoids and CVDs, 9 examined the effect of carotenoid supplementation, and 12 evaluated dietary interventions. **Conclusions:** Elevated serum carotenoid levels are associated with reduced CVD risk factors and inflammatory markers. Increasing the consumption of carotenoid-rich foods appears to be more effective than supplementation, though the specific effects of individual carotenoids on CVD risk remain uncertain.

## 1. Introduction

Coronary artery disease (CAD), which includes angina, myocardial infarction, and heart failure; cerebrovascular disease, which includes stroke and transient ischemic attacks; peripheral arterial disease; and aortic atherosclerosis are the four primary categories of cardiovascular diseases (CVDs). These diseases encompass a range of conditions affecting the heart or blood vessels [1]. Despite advances in prevention and treatment, CVDs remain one of the leading causes of early mortality worldwide, with a 21.1% increase in prevalence observed between 2007 and 2017 [2].

Risk factors for CVDs are divided into non-modifiable and modifiable categories. Non-modifiable risk factors include age (over 55 years), male sex, ethnic origin, family history, and genetic markers [3]. Modifiable risk factors can be further classified as lifestyle factors and metabolic risk factors. Lifestyle factors include inadequate diet, smoking, sedentary behavior, alcohol intake, poor mental health, psychosocial stress, low socioeconomic status, and exposure to environmental toxins. Metabolic risk factors include abnormal blood lipid levels (total cholesterol, triglycerides, high-density lipoprotein, low-density lipoprotein), homocysteine, troponin T, inflammatory markers (*C*-reactive protein, IL-1, IL-6, IL-18, TNF-α, vascular and cellular adhesion molecules), and high blood pressure (BP), among others, as well as the presence of other conditions such as being overweight, obesity, diabetes, and hypertension [4]. Approximately 50% of CVDs are attributable to modifiable risk factors [5].

**Figure 1:**
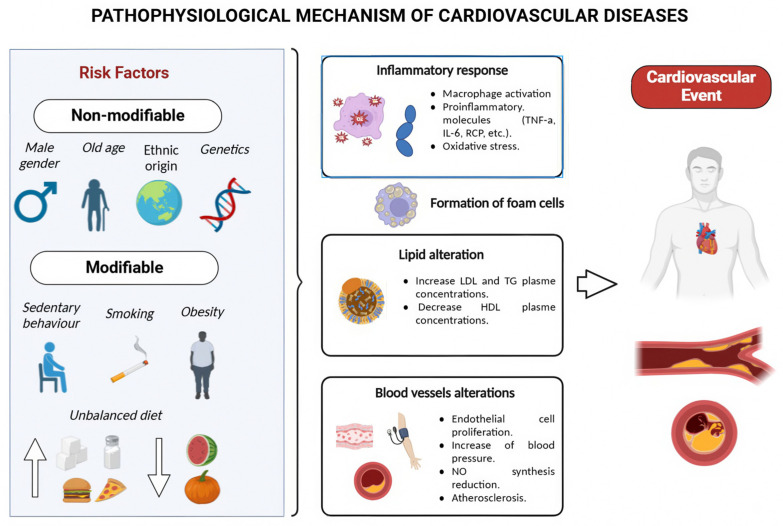
Pathophysiological mechanism of CVDs.

The complex relationship between diet and cardiovascular health has become a central focus in preventive medicine. Higher consumption of fruits and vegetables may reduce the risk of CVDs, lower BP, decrease pro-inflammatory markers, and improve insulin resistance, according to epidemiological studies [6]. The World Cancer Research Fund recommends consuming at least 400 g/day of a variety of non-starchy vegetables and fruits to prevent non-communicable chronic diseases, including CVDs [7]. Phytochemicals, bioactive compounds in addition to traditional macronutrients, appear to play a role in the cardiovascular protective effects of fruits and vegetables [8]. Among these phytochemicals, carotenoids have attracted significant interest for their potential in reducing cardiovascular disease (CVD) risk [9,10] (Figure 1).

Carotenoids are a group of fat-soluble organic pigments synthesized in the chloroplasts of plants, fungi, algae, and some bacteria from an isopentenyl pyrophosphate molecule. As carotenoids consist of eight repeated isoprene units, they are categorized as isoprenoids [11,12].

Based on molecular composition, carotenoids are classified as either carotenes or xanthophylls. Carotenes are hydrocarbon molecules containing only hydrogen and carbon atoms without functional groups. This group includes lycopene, α-carotene, and β-carotene. In contrast, xanthophylls are hydrocarbons that also contain oxygen, including compounds such as β-cryptoxanthin, lutein, zeaxanthin, violaxanthin, neoxanthin, fucoxanthin, astaxanthin, capsanthin, bixin, and crocin [13].

Some carotenoids, including β-carotene, α-carotene, γ-carotene, and β-cryptoxanthin, serve as vitamin A precursors [11]. Vitamin A and its precursors are involved in vision, development, immunity, and the regulation of glucose and fat metabolism [14].

**Figure 2:**
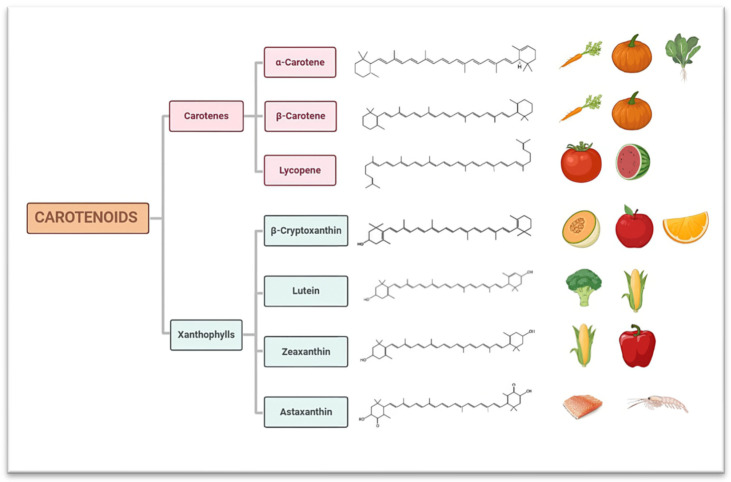
Classification of carotenoids and main sources in Western diet.

Humans cannot synthesize carotenoids, so they must be obtained through diet or supplements [15]. In Western diets, the most commonly consumed carotenoids are α-carotene and β-carotene, found in foods such as carrots, pumpkins, and spinach, and lycopene, present in tomatoes and watermelon. Certain animal foods, such as salmon and crustaceans, can also accumulate carotenoids [16] (Figure 2). The number of carotenoids ingested and their presence in blood plasma are established markers of fruit and vegetable intake [17,18].

It is well known that all carotenoids have strong antioxidant properties that neutralize intracellular reactive oxygen species [19]. By reducing LDL oxidation in cardiovascular tissues, antioxidants contribute to slowing the atherogenesis process. While many carotenoids possess antioxidant qualities, they also improve endothelial function, reduce inflammatory markers, and enhance the lipid profile by mitigating several CVD risk factors through various molecular mechanisms [20].

Lycopene demonstrates anti-inflammatory properties by inhibiting the synthesis of IL-1, IL-6, and TNF-α. It also improves the lipid profile by reducing HDL and triglycerides (TG), inhibits LDL oxidation, and enhances endothelial function while maintaining nitric oxide (NO) levels [21]. Lutein inhibits the transcription of NF-kB and reduces molecules associated with inflammation, such as tumor necrosis factor-alpha (TNF-α), interleukin-6 (IL-6), prostaglandin E2 (PGE-2), monocyte chemoattractant protein-1 (MCP-1), and macrophage inflammatory protein-2 (MIP-2) [22]. It also aids in controlling systolic BP [23].

Recent studies have shown that lutein supplementation lowers some lipid markers, including low-density lipoprotein (LDL), and reduces inflammatory physiological responses. Zeaxanthin regulates the oxidative stress response by lowering oxidized glutathione and increasing intracellular reduced glutathione levels [24]. Higher zeaxanthin levels are also associated with reduced carotid intima-media stiffness (CIMT) [25]. β-carotene decreases LDL oxidation, regulates vascular nitric oxide (NO) bioavailability, lowers nuclear factor kappa-light-chain-enhancer of activated B cells (NF-kB) activation, and reduces the production of pro-inflammatory cytokines, vascular cell adhesion molecule-1 (VCAM-1), intercellular adhesion molecule-1 (ICAM-1), and e-selectin [6].

The objective of this study is to review and synthesize existing literature on the relationship between carotenoids and CVDs. Carotenoids are recognized for their biological activity and antioxidant properties, and they are associated with several physiological processes that influence cardiovascular health. Due to their anti-inflammatory properties and their ability to impact lipid metabolism and endothelial function, carotenoids may offer new avenues for preventing and treating CVDs [26].

This review aims to provide a comprehensive understanding of how carotenoid levels may influence cardiovascular risk factors and promote cardiovascular health by integrating data from epidemiological studies, clinical trials, and mechanistic investigations. By examining the complex relationships between carotenoids and cardiovascular health, this review seeks to provide insights that may inform clinical practice and public health initiatives, paving the way for a more refined approach to managing and preventing cardiovascular disease.

## 2. Materials and Methods

### 2.1. Study Design

This article is a systematic review conducted in accordance with the guidelines outlined in the PRISMA statements [27]. The review aims to answer the following research question: Is there evidence that carotenoid intake can prevent CVDs?

### 2.2. Search Strategy

To identify relevant scientific publications published in English between January 2011 and February 2024, we conducted a comprehensive literature search. A systematic review was performed to gather studies evaluating the effectiveness of carotenoid intake in the prevention of CVDs. The primary databases used in this study were the Cochrane Library and MEDLINE (PubMed).

The search strategy for the MEDLINE database included the following formula: (“carotenoids” OR “carotenoid”) AND (“cardiovascular diseases”) AND (“human” OR “person”) AND (“intervention” OR “study” OR “trial”).

In the Cochrane Library, papers in English were searched using the formula: (“carotenoids” OR “carotenoid”) AND (“cardiovascular diseases”) AND (“human” OR “person”) AND (“intervention” OR “study” OR “trial”).

### 2.3. Selection Criteria

Studies involving subjects aged 18 years or older were selected. Epidemiological observational studies, clinical trials, and randomized controlled trials (RCTs) were included in the analysis.

A detailed list of eligibility criteria, developed using the PICOS (Population, Intervention, Comparison, Outcome, Study Design) format, is presented in Table 1.

### 2.4. Exclusion Criteria

Clinical recommendations, reviews, protocols, preliminary research, and preclinical investigations were not included. Additionally, gray literature was excluded.

### 2.5. Classification of Selected Studies

There are several approaches to studying the effect of carotenoids on cardiovascular health. For this review, studies have been grouped into three main categories: (1) studies that examine the relationship between plasma levels of carotenoids and CVD risk; (2) studies that investigate the effects of oral carotenoid supplementation; and (3) studies that analyze the intake of carotenoids through food or food concentrates. In each case, we assessed the effect of plasma carotenoid levels or carotenoid intake, which are closely related, on CVD markers such as total cholesterol (TC), High-Density Lipoprotein (HDL), LDL, and CIMT values.

## 3. Results

### Study Selection

**Figure 3:**
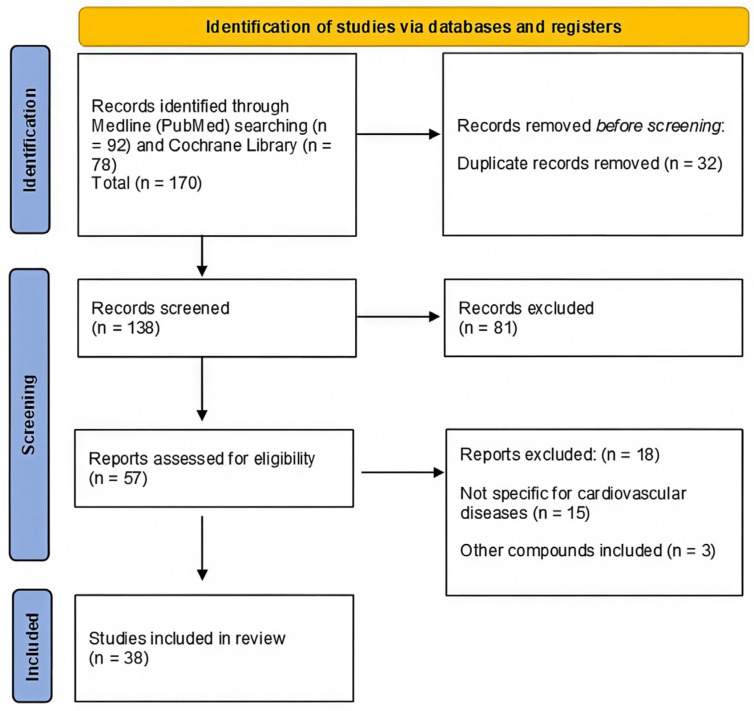
PRISMA flow chart of the systematic review literature search.

As shown in the PRISMA diagram (Figure 3), a total of 170 records were identified from the database searches (PubMed and Cochrane Library). After removing 32 duplicate articles, 138 studies were screened, with 81 excluded based on their titles and abstracts. A total of 57 eligible records underwent full-text screening, and 18 studies were excluded for not meeting the inclusion criteria. At the end of the selection process, 38 papers were included in the qualitative analysis. Seventeen epidemiological observational studies evaluated the relationship between carotenoids and CVDs [25,27,28,29,30,31,32,33,34,35,36,37,38,39,40,41,42], 9 studies examined the effect of carotenoid supplementation on CVDs [43,44,45,46,47,48,49,50,51], and 12 studies evaluated dietary interventions [52,53,54,55,56,57,58,59,60,61,62,63]. Of these, 7 studies provided fruit and vegetable juices [52,56,58,59,60,62,63], and 5 focused on increasing fruit and vegetable consumption [53,54,55,57,61].

The studies included in this review were conducted across various countries, with the highest number performed in the United States (*n* = 9), followed by the UK (*n* = 6), China (*n* = 6), and Spain (*n* = 3). Two studies were conducted in Finland (*n* = 2), Israel (*n* = 2), and Japan (*n* = 2). Australia (*n* = 1), Brazil (*n* = 1), Germany (*n* = 1), the Netherlands (*n* = 1), Norway (*n* = 1), Singapore (*n* = 1), South Korea (*n* = 1), and Sweden (*n* = 1) each contributed one study.

## 4. Discussion

As previously noted, various fruits and vegetables contain carotenoids, which are among the compounds believed to contribute to the health benefits associated with fruit and vegetable consumption. Carotenoids have potential value in new approaches to preventing and treating CVDs due to their anti-inflammatory properties and their ability to influence lipid metabolism and endothelial function [26]. They are particularly promising as a preventive strategy for CVDs, given their widespread presence in the plant kingdom, offering similar health benefits to those of a plant-based or Mediterranean diet [6]. An increase in plasma carotenoid levels is strongly correlated with carotenoid intake, whether through supplementation or consumption of fruits and vegetables [64], making carotenoid consumption a recognized biomarker of fruit and vegetable intake [17].

### 4.1. Observational Epidemiological Studies

The 17 observational studies, summarized in Table 2, examine the relationship between serum carotenoid levels and CVD risk. Some studies indicate that high plasma carotenoid levels are associated with a reduction in CVD risk factors [27,29,30,38,39] and improvements in vascular function [25,27,33]. Furthermore, most of the observational studies reviewed [25,27,28,30,31,32,33,34,35,36,37,38,39,40,41] show that higher plasma carotenoid concentrations, or at least one carotenoid, are linked to a lower risk of developing CVDs, whereas lower carotenoid concentrations are associated with an increased risk [34,35]. Specifically, Prentice RL et al. [31] found that higher levels of α-carotene and β-carotene are associated with lower CVD risk, while lutein and zeaxanthin levels do not appear to affect CVD risk. Huang J, et al. [37] observed a reduced risk of developing CVDs with elevated β-carotene levels. In contrast, Shardell MD, et al. [35] found no association between β-carotene levels and a decreased occurrence of all-cause disease; only higher lycopene levels were related to a lower risk of developing CVDs and all-cause mortality. Matsumoto M. et al. [39] showed that maintaining high blood concentrations of total carotenoids is inversely associated with seven CVD risk biomarkers in men (brachial-ankle pulse wave velocity, systolic BP, diastolic BP, insulin resistance index (HOMA-IR), insulin, and HDL). Zhu X. et al. [40] examined the correlation between serum carotenoids and cardiovascular and all-cause mortality risk and found that lower levels of α-carotene, β-cryptoxanthin, and lycopene were linked to lower cardiovascular mortality, although β-carotene and lutein/zeaxanthin were not. Wang M. et al. [49] reported that the prevalence of CVDs, particularly heart attack and stroke, was inversely correlated with blood levels of lutein/zeaxanthin, α-carotene, lycopene, and β-cryptoxanthin. Lastly, Wang Y. et al. [34] identified a strong negative correlation between homocysteine levels and dietary intake of β-carotene, lycopene, and total carotenoids, as well as an inverse association between LDL serum levels and dietary intake of β-carotene and lutein/zeaxanthin. Additionally, they observed a positive correlation between HDL concentrations and dietary lutein/zeaxanthin intake.

Regarding cardiovascular function, Zou Z. et al. [25] found that lower serum lutein levels were associated with a higher CIMT, while lower levels of zeaxanthin and β-carotene were linked to increased stiffness in the right common carotid artery. Wang C. et al. [33] observed an inverse, dose-dependent association between serum carotenoid concentrations and mean CIMT. Among the carotenoids studied, β-carotene showed the strongest association, followed by total carotenoids, lutein/zeaxanthin, α-carotene, and β-cryptoxanthin, with lycopene exhibiting the weakest association. Huang Y. et al. [27] found that serum levels of α-carotene, all-trans-β-carotene, and lycopene were independently associated with higher heart rate variability (HRV).

One study [36] that examined the relationship between blood carotenoids and IL-6 levels, an inflammatory marker in patients with CAD, found that only lutein/zeaxanthin showed an inverse correlation with IL-6 in individuals with stable angina. The other carotenoids included in the study showed no correlation with lutein/zeaxanthin.

Some studies examined the relationship between plasma carotenoid levels and hypertension as an indicator of CVDs. Hozawa A. et al. [30] found that lycopene was not correlated with hypertension in any model, but four carotenoids—lutein/zeaxanthin, β-carotene, β-cryptoxanthin, and α-carotene—were significantly inversely correlated with hypertension. Toh DWK et al. [38] showed that plasma carotenoid levels were inversely associated with systolic and diastolic BP. Matsumoto M. et al. [39] reported similar results.

However, some authors have not found a relationship between carotenoid levels and a reduction in CVD risk and, in some cases, have observed an increase in certain markers. For example, Wang L. et al. [29] found that in middle-aged and older women, an increase in LDL was associated with higher plasma levels of α-carotene, β-carotene, and lycopene; an increase in HDL was associated with lower plasma lycopene levels; and an increase in CRP was associated with lower plasma β-carotene concentrations. Matos A. et al. [32] did not observe any significant correlation between β-carotene levels and CVDs. Additionally, Qiu Z. et al. [42] reported that, while other individual carotenoids (α-carotene, β-cryptoxanthin, lycopene, and lutein/zeaxanthin) were not significantly associated with cardiovascular mortality, higher serum β-carotene concentrations were significantly associated with an increased risk of cardiovascular mortality. It should be noted, however, that this study was conducted in individuals with type 2 diabetes.

Except for three studies [29,32,42], most of the recent observational studies generally indicate a correlation between blood carotenoid concentrations and a protective effect against CVDs. The most consistent correlations are observed with total carotenoid levels [30,33,35,38,39,40,41], though findings for specific carotenoids are more variable [25,27,29,30,31,32,34,35,37].

Most of these observational studies do not clarify whether the reduction in mortality is directly due to carotenoid effects, whether other dietary components in fruits and vegetables play a role in CVD development, or whether carotenoid intake is from supplements or carotenoid-rich foods.

Given that high fruit and vegetable intake is widely associated with elevated serum carotenoid levels, it remains uncertain whether carotenoids alone prevent CVDs or if they interact with other components of fruits and vegetables to produce these effects. Consequently, it would be valuable to examine findings from studies that provide carotenoid supplements to assess their effectiveness in CVD prevention.

### 4.2. Intervention Studies with Carotenoid Supplements

There are nine studies that examine the effect of carotenoid supplements on CVDs (Table 3). Gajendragadkar P. et al. [43] investigated the effects of two months of lycopene supplementation in patients with CVDs and in healthy volunteers. They found that lycopene improved endothelial function in patients with CVDs but had no effect on age-matched healthy volunteers. Wolak T. et al. [45] evaluated the effect of capsules containing a tomato nutrient complex with 15 mg and 30 mg of lycopene, comparing it to capsules with 15 mg of synthetic lycopene and a placebo. The study found that the tomato nutrient complex, containing both 15 mg and 30 mg of lycopene, effectively lowered systolic BP in healthy volunteers, whereas lower doses of pure lycopene did not produce comparable benefits. No statistically significant effect was observed on diastolic BP. Zou Z. et al. [47] assessed the effects of supplementing individuals with subclinical atherosclerosis with lutein (20 mg) alone and in combination with lycopene (20 + 20 mg). The combined lutein and lycopene treatment was more effective in preventing CIMT progression, while both treatments reduced CIMT. Xu XR et al. [44] examined the effects of three months of lutein supplementation (20 mg) on early atherosclerosis, focusing on inflammation biomarkers associated with CVDs (IL-6, MCP-1) and lipid profile (LDL, TG). They observed significant reductions in serum TG, LDL levels, and inflammatory cytokines. Stonehouse W. et al. [48] investigated the effects of an eight-week carotene supplementation (21 mg daily) on vascular function and CVD risk factors in adults at high risk of poor vascular function. No significant effects were found on vascular function, as measured by brachial artery flow-mediated dilatation, pulse wave velocity, atherogenic index, or circulating markers of vascular function. Additionally, no notable impact was observed on other CVD risk factors, including inflammatory markers, lipid profiles, BP, or glucose metabolism.

Kawashima A. et al. [49] examined the effects of four weeks of supplementation with a capsule containing dehydrated concentrates from various fruit and vegetable juices on DNA damage, oxidative stress indicators, and plasma homocysteine levels. While serum lipid peroxide levels decreased following the intervention, these results did not differ significantly from those of a placebo. Engelhard Y. et al. [50] administered one capsule daily of encapsulated tomato extract to subjects with grade 1 hypertension over eight weeks and found a significant reduction in both systolic and diastolic BP, as well as a decrease in lipid peroxidation products. However, the supplement did not affect blood lipids, lipoproteins, or homocysteine levels. Graydon R. et al. [56] conducted an eight-week intervention with middle-aged, healthy subjects who received either a mixed lutein and zeaxanthin supplement (containing 5 mg zeaxanthin and 10 mg lutein), a 15 mg β-carotene supplement, or a placebo. No changes were observed in macular pigment level (MPL) or in markers of endothelial activation, inflammation, or oxidation following supplementation with either spinach powder or carrot juice. However, an improvement in MPL was noted in the highest serum responders and in those with initially low MPL. Ryu N. et al. [51] evaluated the effects of 5 g per day of Chlorella powder tablets versus lactose powder tablets over four weeks in subjects with mild hypercholesterolemia. The Chlorella group showed significant reductions in total cholesterol and triglycerides. Improvement in serum lipids was supported by notable decreases in very low-density lipoproteins (VLDL), apolipoprotein, non-HDL, and the HDL/TG ratio, suggesting an inhibitory effect of Chlorella on the intestinal absorption of dietary and endogenous lipids. The authors propose that these changes in serum lipids were related to changes in serum carotenoid levels.

Lastly, Schwab S. et al. [46] investigated the association between carotenoid supplement use and changes in HbA1c levels, a biomarker of CVDs, over ten years in non-diabetic individuals. They found that carotenoid intake above 6.8 mg/day was associated with a smaller increase in HbA1c levels compared to no carotenoid intake, but this effect was observed only in non-smokers.

Overall, it appears that combinations of carotenoids [45,46,47,50,51] are more effective than supplementing a single carotenoid [43,44], although the results are generally more modest than those observed in epidemiological studies, as the same carotenoids are not always supplemented and do not consistently produce the expected effect. However, at comparable dosages to those used in other studies, two studies [48,49] reported no effect of carotenoid supplementation.

### 4.3. Dietary Intervention Studies

Finally, 12 studies that utilized dietary interventions to increase carotenoid consumption were analyzed: seven studies provided vegetable and fruit juices (Table 4), and five studies focused on increasing fruit and vegetable intake (Table 5).

#### 4.3.1. Vegetable and Fruits Juices

Takagi T. et al. [52] analyzed the effects of an eight-week intake of a vegetable drink rich in carotenoids, specifically lycopene or lutein, in men with a body mass index (BMI) over 25 kg/m^2^. They observed a reduction in visceral adiposity and oxidative stress, specifically in the CoQ10 oxidation rate. Colmán-Martínez M. et al. [58] investigated the effects of providing subjects at high risk for CVDs with either 200 mL or 400 mL of tomato juice or a placebo for four weeks. They found that trans-lycopene from tomato juice may reduce CVD risk by lowering the concentration of inflammatory molecules associated with atherosclerosis, such as adhesion molecules ICAM-1, VCAM-1, and IL-8. These markers were not significantly reduced by other carotenoids. Paterson E. et al. [59] studied the effect of consuming one soup (500 mL) plus one juice (300 mL) or one shot (100 mL of a concentrated fruit and vegetable preparation) daily for one month in healthy subjects. They observed a significant reduction in plasma homocysteine concentrations following the dietary intervention, although other risk markers remained unaffected. Bub A. et al. [60] examined the effects of daily consumption of 330 mL of tomato juice, followed by 330 mL of carrot juice and 10 g of spinach powder, each consumed for two weeks in healthy men. The results showed that tomato juice reduced plasma thiobarbituric acid reactive substances and lipoprotein oxidisability by increasing lag time, whereas carrot juice and spinach powder had no effect on lipid peroxidation. Additionally, water-soluble antioxidants, ferric-reducing antioxidant power (FRAP), and activities of glutathione peroxidase and reductase remained unchanged throughout the study periods. Biddle M. et al. [63] supplemented 11.5 ounces of low-sodium vegetable juice daily for one month in patients with heart failure and found no difference in CRP levels between the control and intervention groups. Tomás A. et al. [62] observed statistically significant changes in serum levels of LDL, VLDL, HDL, triglycerides, and atherogenic index after administering orange–carrot juice, tomato juice, and boiled spinach for four weeks to perimenopausal and postmenopausal women with cardiometabolic risk factors but no prior CVDs symptoms. Lastly, Graydon R. et al. [56] conducted an eight-week intervention with middle-aged, healthy subjects who consumed either 131 mL of carrot juice or 10.4 g of dried spinach powder daily. No changes were observed in macular pigment levels (MPL) or in markers of endothelial activation, inflammation, or oxidation following supplementation with spinach powder or carrot juice. However, MPL improvements were noted in the highest serum responders and those with initially low MPL.

Contradictory findings are apparent regarding the impact of carotenoid consumption through fruit and vegetable juices. Effects tend to be more noticeable in individuals with existing cardiovascular symptoms or risk factors [52,58,62]. Although some studies found no impact [63], certain studies have shown minor benefits on inflammatory markers in healthy individuals [59,60]; however, Graydon R. et al. [56] identified improvements only in those with low baseline carotenoid levels (Table 4).

#### 4.3.2. Increased Consumption of Fruit and Vegetable

In this section, we review studies that have directly increased fruit and vegetable consumption as an intervention. Daniels J. et al. [53] randomly assigned obese subjects with type 2 diabetes to a diet of either one or six or more portions of fruits and vegetables daily for eight weeks. They found that the carotenoid content in HDL2 and HDL3 increased, especially for α-carotene, β-cryptoxanthin, lutein, and lycopene in HDL3, indicating an enhancement in HDL’s antioxidant properties. Wallace I. et al. [54] studied the effects of consuming one, two, four, or seven portions of fruits and vegetables per day over 12 weeks in subjects with a CVD risk of 20% or higher and a mean age of 56 years but found no significant changes in whole-body, peripheral, or hepatic insulin resistance (IR) or adiponectin multimers. Svendsen M. et al. [61] examined the effects of consuming 400 g/day of vegetables and 300 g/day of fruit over three months in obese subjects with sleep-related breathing disorders. The intervention group showed reductions in weight and BP but no changes in antioxidant defences, as measured by FRAP. In contrast, Hurtado-Barroso S. et al. [55] investigated the acute effects of a single dose of sofrito sauce (240 g/70 kg body weight) in fasting healthy volunteers. They observed improvements in inflammatory biomarkers, specifically CRP and TNF-α, although IL-6 was unaffected. An inverse relationship was found between β-carotene levels and the reduction in TNF-α. Lastly, Thies F. et al. [57] analyzed the effects of a low tomato-based foods diet, a high tomato-based diet, or a control diet supplemented with lycopene capsules (10 mg/day) for 12 weeks in healthy, middle-aged subjects. They found no changes in systemic markers (CRP, IL-6, ICAM-1, oxidized LDL, HOMA-IR) or in the quantitative insulin-sensitivity check index after the dietary intervention. Additionally, lipid concentrations and arterial stiffness were unaffected by the interventions.

There is ongoing debate over the impact of increasing the consumption of carotenoid-rich fruits and vegetables, as some research has yielded inconclusive results [54,57]. While some studies have reported reductions in weight and BP in obese individuals [61], most research has examined improvements in antioxidant capacity, showing that higher carotenoid intake leads to significant improvements [53,55] (Table 5).

### 4.4. Final Analysis

**Figure 4:**
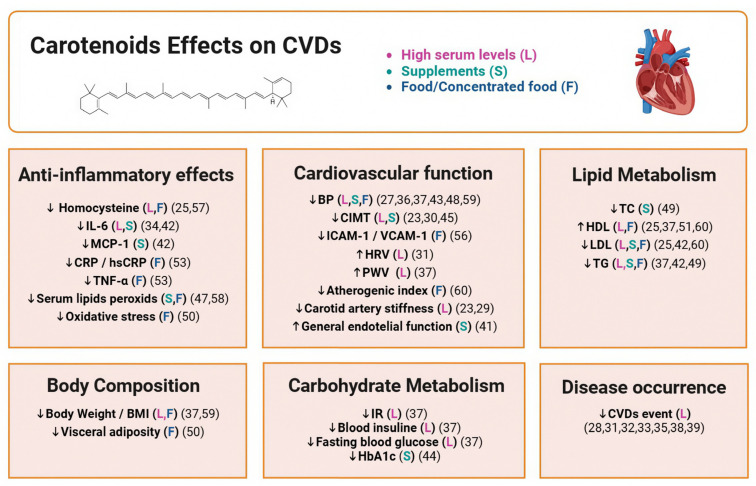
Summary of the effects of the carotenoids on CVDs.

In summary, epidemiological research has shown more promising results than intervention studies. Key findings regarding the impact of carotenoid intake on CVDs are compiled in Figure 4. Unlike intervention studies, which typically involve short-term interventions lasting one to three months, epidemiological studies span from one to twenty years and consider long-term dietary patterns, so some observed outcomes may be time-dependent [25,27,29,30,31,32,33,34,35,37,38,39,40,41,42]. An exception to this trend is the study by Schwab S. et al. [46], which examined the effects of carotenoid supplementation over a ten-year period and found a cardioprotective effect, and the study by Zou Z. et al. [47], which demonstrated that lutein or lutein/lycopene supplementation can effectively prevent CIMT progression. This suggests that the timing of carotenoid intake assessment may be critical in evaluating its impact on CVD risk indicators and actual risk reduction. Several studies examining short-term antioxidant capacity [44,53,55,59,60] have yielded encouraging results, suggesting that sustained intake over time could translate into physiological benefits. However, short-term effects appear more pronounced in individuals with existing CVDs than in healthy individuals [43,44,45,50,51,52]. Based on the reviewed data, the combined effect of all carotenoids appears to offer greater cardiovascular benefits than any single carotenoid. In fact, studies supplementing with a single carotenoid [43,45,47] tend to show less favourable outcomes than those using multiple carotenoids or a single plant extract [45,46,50,51]. Most clinical trials included in this review are short in duration, and although improvements in controlling CVD risk factors or inflammation markers are observed, the extent to which these improvements reduce the risk of developing CVDs remains unclear, as none of the clinical trials have directly assessed this relationship. It is also important to note that each study evaluates different carotenoids or combinations of carotenoids, and supplement dosages vary, making it challenging to compare findings across studies.

### 4.5. Limitations of the Study

This review is limited by the well-known variability in clinical trials, which affects both the carotenoids examined and the CVD risk factors assessed. The comparison of the effects on CVD risk reduction between dietary supplements, carotenoid-rich foods, and concentrated carotenoid sources is particularly challenging due to the different carotenoid’s combinations, and the significant differences in de dosages of carotenoids used in these interventions. Moreover, many of the included studies do not account for baseline consumption of fruits and vegetables, both before and during the intervention period, nor do they assess the overall diet quality of participants. These dietary factors may contain additional bioactive compounds that interact with carotenoids and, therefore, could collectively influence the reduction of CVD risk factors.

Another limitation is the lack of clarity regarding the molecular mechanisms through which carotenoids contribute to the reduction of various CVD risk factors. This gap complicates the ability to establish definitive conclusions about the biological pathways involved.

Moreover, although some studies have found reductions in the risk factors for CVDs, it is challenging to ascertain if these modifications result in long-term decreases in the incidence of CVDs due to the brief duration of interventions.

Future studies should address the gaps by standardizing carotenoids combinations and dosages and sources to allow for more accurate comparisons of their effects on CVD risk factors. Longer intervention periods are necessary to assess the long-term impact of carotenoids on actual CVD incidence. Additionally, comprehensive dietary assessments, including baseline fruit and vegetable intake and overall diet quality, should be incorporated to better isolate the effects of carotenoids. Research should also explore the molecular mechanisms through which carotenoids influence CVD risk and include diverse populations to determine their efficacy across different demographic groups. Finally, RCTs with larger sample sizes and the investigation of synergistic effects with other nutrients are needed to strengthen the evidence and provide more definitive conclusions.

## 5. Conclusions

This review provides an overview how carotenoids intake from diet and supplements affects the development and course of CVDs. Elevated blood carotenoid levels are associated with reduced CVD risk factors and inflammatory markers, potentially lowering the likelihood of cardiovascular events. Increasing the consumption of carotenoids rich foods seems more effective than supplementation in reducing inflammatory markers and CVD risk indicators. Natural sources of carotenoids, which contain a combination of carotenoids and other phytocompounds, may enhance or modify the effects of carotenoids on CVD risk. Consequently, the effect of individual carotenoids on CVD risk remains unclear. Additionally, the short duration of most clinical trials limits the ability to assess the long-term impact of carotenoids intake on CVD risk reduction. Despite these limitations, it is evident that consuming a diverse range of carotenoids, mostly through food, may delay the onset and progression of CVDs.

As already mentioned in the limitations of the study, future research should focus on longer-term clinical trials to explore the direct relationship between carotenoid intake and the actual occurrence of CVDs, going beyond the mere assessment of risk factors or markers.

## Figures and Tables

**Table 1:** PICOS table for inclusion of studies.

Parameters	Inclusion Criteria
Population	Age 18 years or older
Intervention	Carotenoid intake as CVD prevention
Comparison	Low carotenoids intake
Outcomes	Health and disease markers
Study design	Epidemiological observational studies, clinical trials, and RCTs

**Table 2:** Observational studies about carotenoids and CVDs.

Author, Publication Year	Country/Region	Type of Study/Study Name	Follow-Up Period	Study Size	Carotenoids Evaluated	Findings
Wang, L. et al., 2008 [29]	USA	Case-control studies/Women’s Health Study	1 year and 6 months	39,876	α-carotene, β-carotene, β-cryptoxanthin, lycopene, and lutein/zeaxanthin	Association of ↑ α-carotene, β-carotene, and lycopene with ↑ LDL. ↓ lycopene with ↑ of HDL and ↑ of HbA1c. ↓ β-carotene with ↑ of CRP.
Hozawa, A. et al., 2009 [30]	USA	Prospective, multicentre epidemiologic study/ Coronary Artery Risk Development in Young Adults Study	20 years	4412	α-carotene, β-carotene, lutein/zeaxanthin, cryptoxanthin, lycopene	Sum of 4 carotenoids was significantly inversely associated with HT. Lycopene was unrelated to HT in any model.
Prentice, RL. et al., 2019 [31]	USA	Randomized controlled Clinical Trial/Nutrition and Physical Activity Assessment Study	5 years	5488	α- and β-carotene, lutein + zeaxanthin (L+Z), and α-tocopherol	↑ levels of α-carotene, β-carotene with ↓ risk of CVDs. ↑ levels of L+Z shown not to affect CVDs.
Matos, A. et al., 2018 [32]	Brazil	Cross-sectional observational study	1 year	90	β-carotene	β-carotene diminished as the extent score rose of CAD, although this was not statistically significant.
Zou, Z. et al., 2011 [25]	China	Case-control study	Baseline	125	Lutein, zeaxanthin, β-carotene and lycopene	↓ levels serum lutein with ↑ CIMT. ↓ levels serum Zeaxanthina and β-carotene with ↑ carotid artery stiffness.
Wang, C. et al., 2018 [33]	China	Cross-sectional study/ Guangzhou Nutrition and Health Study	Baseline	2947	α-carotene, β-carotene, lutein + zeaxanthin, β-cryptoxanthin and lycopene	↑ carotenoid levels in diet and serum are associated with lower carotid CIMT values
Huang, Y. et al., 2021 [27]	USA	Cross-sectional analysis/Midlife in the United States	Baseline	1074	Lutein, zeaxanthin, β-cryptoxanthin, 13-cis-β-carotene, α-carotene, all-trans-β-carotene and lycopene	Blood α-carotene, all-trans-β-carotene and lycopene levels were independently associated with higher HRV, reducing the risk of CVDs.
Karppi, J. et al., 2012 [34]	Finland	Prospective cohort study/Kuopio Ischaemic Heart Disease Risk Factor	15.9-year follow-up	1031	Lycopene, α-carotene, β-carotene	Low serum concentrations of β-carotene were strongly related to an increased CVDs mortality risk after adjustment for confounders.
Shardell, D. et al., 2011 [35]	USA	Observational study, NHANES III	14.3 years	13,293	α-carotene, β-carotene, β-cryptoxanthin, lycopene, and lutein + zeaxanthin	Low α-carotene was associated with ↑ CVDs mortality. Very low serum of total carotenoid, α-carotene, and lycopene concentrations may be risk factors for mortality.
Chung, RWS. et al., 2017 [36]	Sweden	Cross-sectional and longitudinal study	3 months	193	Lutein + zeaxanthin, β-cryptoxanthin, lycopene, α- and β-carotene and IL-6	Inverse association between lutein and IL-6 in CAD patients.
Huang, J. et al., 2018 [37]	Finland	Prospective Cohort Study	5–8 years between 1985 and 1988	29,133	β-carotene	Higher β-carotene biochemical status is associated with lower overall CVDs, heart disease, stroke, and other causes of mortality.
Toh, DWK. et al., 2021 [38]	Singapore	Cross-sectional study	13 months	108	β-carotene, α-carotene, lycopene, lutein, zeaxanthin and β-cryptoxanthin	Skin carotenoids and plasma carotenoids were inversely associated with systolic BP and diastolic BP.
Matsumoto, M. et al., 2020 [39]	Japan	Resident-based cross-sectional study	Baseline	1350	Lutein, zeaxanthin, β-cryptoxanthin, α-carotene, β-carotene, and lycopene	Higher concentration of serum carotenoids in relatively healthy individuals was associated with better CVD markers.
Zhu, X et al., 2023 [40]	China	Prospective study NHANES III	6 years	13,688	Lutein/zeaxantine, α-carotene, β-carotene, β-cryptoxanthin, lycopene	Higher concentrations of major serum carotenoids were associated with decreased risk of cardiovascular mortality.
Wang, M et al., 2023 [41]	China	Cross-sectional study	5 years	12,424	Lutein/zeaxantine, α-carotene, β-carotene, β-cryptoxanthin, lycopene	Serum carotenoids were negatively associated with the prevalence of CVDs.
Wang, Y. et al., 2014 [28]	USA	Cross-sectional study	3 years	2856	Individual dietary carotenoid intake	Significant inverse associations with LDL cholesterol were observed for dietary β-carotene and lutein + zeaxanthin, and with homocysteine for dietary β-carotene, lycopene and total carotenoids. Dietary lutein + zeaxanthin intake was also positively associated with HDL concentrations.
Qiu, Z. et al., 2022 [42]	USA	Prospective study	5 years (2001–2006)	3107	α-carotene, β-carotene, β-cryptoxanthin, lutein/zeaxanthin, and lycopene	The action of β-carotene on people with type 2 diabetes is unclear.

**Table 3:** Intervention studies with carotenoid supplementation.

Author, Publication Year	Country/ Region	Type of Study/Study Name	Follow-Up Period	Study Size	Carotenoids Supplemented	Findings
Gajendragadkar, Pr. et al., 2014 [43]	UK	Randomized, double-blind trial	2 months	72	Lycopene	Lycopene supplementation improves endothelial function in CVDs but not in healthy volunteers.
Xu, XR et al., 2013 [44]	China	Randomized, double-blind, placebo-controlled intervention trial	3 months	65	Lutein	After 3 months of supplementation with lutein ↓ IL-6, MCP-1, LDL, and TG levels.
Wolak, T. et al., 2019 [45]	Israel	Double-blind, randomized, placebo-controlled study	2 months	61	Tomato nutrient complex (5, 15 and 30 mg lycopene) vs. 15 mg of synthetic lycopene	Carotenoid levels achieved by the tomato nutrient complex (TNC) dose of 15 mg lycopene or higher correlate to a beneficial effect on systolic BP in hypertensive subjects, while lower doses and lycopene alone do not.
Schwab, S. et al., 2015 [46]	Germany	Two population-based cohorts/Monitoring of Trends and Determinants in Cardiovascular Diseases and Cooperative Health Research in the Region of Augsburg	10 years	2774	Carotenes	High carotenoid intake could be one strategy for the prevention of cardiovascular complications in non-diabetic people. ↓ HbA1c levels.
Zou, Z. et al., 2014 [47]	China	Randomized, double-blind, placebo-controlled trial.	12 months	144	Lutein and lycopene	The mean values of CAIMT decreased significantly in the lutein and combination groups at month 12. The change in CIMT was inversely associated with the increase in serum lutein concentrations in both the active treatment groups and with that in serum lycopene concentrations in the combination group.
Stonehouse, W. et al., 2016 [48]	Australia	A randomized, placebo-controlled, double-blind study	2 months	90	Carotenes	Carotenes had no effects on vascular function or CVD risk factors.
Kawashima, A. et al., 2007 [49]	USA	Double-blinded placebo controlled randomized study	1 month	60	Juice	Serum lipid peroxides and urine concentrations of 8-OHdG decreased significantly but were not significantly different than a placebo.
Engelhard YN. et al., 2006 [50]	Israel	Double-blinded, placebo-controlled pilot study	8 weeks	31	Tomato extract	Reduced systolic and diastolic BP in patients with grade 1 hypertension. No significant changes were found in lipid parameters.
Ryu, NH. et al., 2014 [51]	South Korea	Double-blinded, randomized, placebo-controlled study	4 weeks	63	5 g Chlorella powder a day	Chlorella group exhibited remarkable changes in TC, TG, lutein/zeaxanthin, and α-carotene.

**Table 4:** Intervention studies with dietetic carotenoids providing vegetable and fruit juices.

Author, Publication Year	Country/Region	Type of Study/Study Name	Follow-Up Period	Study Size	Dietary Intervention	Findings
Takagi, T. et al., 2020 [52]	Japan	Randomized, double-blinded, controlled clinical trial	8-weeks	28	High lycopene + high lutein, high lycopene + low lutein, low lycopene + high lutein, and low lycopene + low lutein by vegetable beverages	Daily beverage-intake significantly decreased the visceral fat level, and CoQ10 oxidation rate was decreased in all the groups.
Graydon, R. et al., 2012 [56]	UK	Randomized placebo-controlled trial	8-week	52	Dried spinach powder (lutein and zeaxanthin-rich food) or carrot juice (α and β-carotene rich food)	Lutein and zeaxanthin had no significant effect on MPL or serological markers of endothelial activation, inflammation and oxidation in healthy volunteers.
Colmán-Martínez, M. et al., 2017 [58]	Spain	Retrospective, randomized, cross-over, and controlled clinical trial	4 weeks	28	200 mL (LD) or 400 mL (HD) of tomato juice	Trans-lycopene reduced the concentration of important adhesion molecules ICAM-1, and VCAM-1, related to atherosclerosis.
Paterson, E. et al., 2006 [59]	UK	Single blind, randomized, controlled, crossover dietary intervention study.	4 weeks	36	During the test intervention period, the subjects were asked to consume 1 soup (500 mL) plus 1 juice (300 mL) or shot (fruit and vegetable preparation made from concentrated juices and purees) (100 mL) per day	Consumption of the carotenoid-rich soups and beverages only decreased the plasma homocysteine concentration by 8.8%.
Bub, A. et al., 2000 [60]	The Netherlands	Clinical trial	8 weeks	23	330 mL/d of a tomato juice (40 mg lycopene) in addition to their meals or 330 mL carrot juice (15.7 mg a-carotene and 22.3 mg b-carotene) daily	Tomato juice consumption reduced plasma thiobarbituric acid reactive substances (TBARS) and lipoprotein oxidizability in terms of an increased lag time. Carrot juice and spinach powder had no effect on lipid peroxidation.
Tomás, A. et al., 2021 [62]	Spain	Clinical trial	4 weeks	12	Orange-carrot juice, tomato juice, and boiled spinach, providing 415 mg of total carotenoids/week (carotenes, cryptoxanthin, lycopene, and lutein + zeaxanthin)	Significant decrease in LDL and atherogenic index, and an increase in HDL were observed.
Biddle, MJ. et al., 2015 [63]	USA	Two-group, randomized controlled intervention pilot study	30 days	40	11.5 ounces of a juice of vegetables (29.4 mg of lycopene; 70 calories; 140 mg of sodium; vitamins A and C; 820 mg of potassium; 2% of the recommended daily allowance for iron and magnesium; and 3 g of fiber	No differences on CRP levels.

**Table 5:** Intervention studies with dietetic carotenoids by increasing fruit and vegetable intake.

Author, Publication Year	Country/Region	Type of Study/Study Name	Follow-Up Period	Study Size	Dietary Intervention	Findings
Daniels, JA. et al., 2014 [53]	UK	Randomized, double-blinded, controlled clinical trial	8-weeks	80	Randomized to a 1- or ≥6-portion/day fruit and vegetables diet	≥6- vs. 1-portion post-intervention comparisons, carotenoids increased in serum, HDL2 and particularly HDL3, as did the activities of PON-1 and LCAT in HDL3.
Wallace, I. et al., 2013 [54]	UK	Randomized controlled trial	12-week	89	One to two, four, or seven portions of FandVs	No significant difference was found in measures of whole-body, peripheral, or hepatic IR or adiponectin multimers.
Hurtado-Barroso, S. et al., 2019 [55]	Spain	Clinical trial	1 day	22	Single portion of sofrito (240 g/70 kg bodyweight) in a state of fasting	Significant decrease in CRP and TNF-α was observed, but only TNF-α was inversely correlated with an increase in TPE (total polyphenol excretion) and plasma β-carotene.
Thies, F. et al., 2012 [57]	UK	Single-blind, randomized controlled trial	12 weeks	225	Diet low in tomato-based foods, a high-tomato-based diet, or a control diet supplemented with lycopene capsules (10 mg/d)	High daily consumption of tomato-based products or lycopene supplements is ineffective at reducing conventional CVD risk markers.
Svendsen M. et al., 2007 [61]	Norway	Randomized, controlled trial	3 months	138	Consumption of vegetables to at least 400 g/day, and fruit to at least 300 g/day	Weight reduction and reduced systolic and diastolic BP. No effect on antioxidant defence measured with FRAP.

## Data Availability

Not applicable.
